# Novel antiviral approaches for Marburg: a promising therapeutics in the pipeline

**DOI:** 10.3389/fmicb.2024.1387628

**Published:** 2024-04-25

**Authors:** Shriyansh Srivastava, Sachin Kumar, Sumel Ashique, Sathvik Belagodu Sridhar, Javedh Shareef, Sabin Thomas

**Affiliations:** ^1^Department of Pharmacology, Delhi Pharmaceutical Sciences and Research University (DPSRU), New Delhi, India; ^2^Department of Pharmacy, School of Medical and Allied Sciences, Galgotias University, Greater Noida, India; ^3^Department of Pharmaceutical Sciences, Bengal College of Pharmaceutical Sciences & Research, Durgapur, West Bengal, India; ^4^RAK College of Pharmacy, RAK Medical & Health Sciences University, Ras al Khaimah, United Arab Emirates; ^5^Clinical Pharmacy & Pharmacology, RAK College of Pharmacy, RAK Medical & Health Sciences University, Ras al Khaimah, United Arab Emirates; ^6^College of Health Sciences, University of Nizwa, Nizwa, Oman

**Keywords:** Marburg virus disease, antiviral therapy, small molecule inhibitors, RNA interference, immune modulators, clinical trials, combination therapy, drug resistance

## Abstract

Marburg virus disease (MVD) presents a significant global health threat, lacking effective antivirals and with current supportive care offering limited therapeutic options. This mini review explores the emerging landscape of novel antiviral strategies against MVD, focusing on promising therapeutics currently in the development pipeline. We delve into direct-acting antiviral approaches, including small molecule inhibitors targeting viral entry, replication, and assembly, alongside nucleic acid antisense and RNA interference strategies. Host-targeting antivirals are also considered, encompassing immune modulators like interferons and cytokine/chemokine modulators, broad-spectrum antivirals, and convalescent plasma and antibody-based therapies. The paper then examines preclinical and clinical development for the novel therapeutics, highlighting *in vitro* and *in vivo* models for antiviral evaluation, safety and efficacy assessments, and the critical stages of clinical trials. Recognizing the challenges of drug resistance and viral escape, the mini review underscores the potential of combination therapy strategies and emphasizes the need for rapid diagnostic tools to optimize treatment initiation. Finally, we discuss the importance of public health preparedness and equitable access to these promising therapeutics in achieving effective MVD control and global health security. This mini review presents a comprehensive overview of the burgeoning field of MVD antivirals, highlighting the potential of these novel approaches to reshape the future of MVD treatment and prevention.

## 1 Introduction

Marburg virus disease, a highly virulent illness causing hemorrhagic fever, has a fatality ratio of up to 88%. It belongs to the same family as Ebola virus disease (WHO, 2024). The disease is rare but severe, affecting both humans and non-human primates, and spreads through contact with bodily fluids. Symptoms include fever, headache, malaise, muscle aches, diarrhea, abdominal pain, vomiting, and neurological involvement (WHO, 2024). Treatment primarily involves supportive hospital therapy as there is no specific treatment available (CDC, 2021), while prevention measures focus on avoiding contact with infected bodily fluids (CDC, 2023). The treatment landscape for Marburg disease faces limitations due to limited understanding of its pathogenesis and lack of medical equipment in affected areas (Rivera and Messaoudi, 2015; Sword et al., 2023). The high lethality and low number of cases further impede treatment development (Sword et al., 2023). Currently, there are no approved specific antiviral treatments for Marburg virus, but promising research suggests potential with drugs like T-705 (Favipiravir) and monoclonal antibody regimens (Zhu et al., 2018; Bradfute, 2022). Development of effective treatments and vaccines is crucial to combat this deadly virus (Bradfute, 2022).

## 2 Emerging therapeutic approaches for Marburg virus infection

Various ongoing studies have identified promising antiviral strategies for Marburg virus, including the development of vaccines, investigation of antiviral drugs, antibodies, and identification of potential compounds targeting specific viral proteins. In its first-in-human clinical trial, researchers at the National Institute of Allergy and Infectious Diseases (NIAID) developed an experimental Marburg virus vaccine called cAd3-Marburg, which has shown promising results. Using a modified chimpanzee adenovirus, the vaccine successfully induced immune responses against the Marburg virus. It was determined to be safe and effectively triggered an immune response in participants, with 95% exhibiting a robust antibody response post-vaccination, and 70% maintaining this response for over 48 weeks. Further trials of the cAd3-Marburg vaccine are planned in multiple countries, including Ghana, Kenya, Uganda, and the United States (Health NTDI, 2023). Estradiol benzoate and INVEGA (paliperidone) demonstrate potential as inhibitors of the VP35 protein of the Marburg virus, identified through a cheminformatics approach. The VP35 protein plays a crucial role in the virus’s replication and immune evasion. These compounds exhibit favorable binding free energies, indicating their potential to disrupt VP35 function, thereby hindering viral replication and immune evasion. However, experimental validation is necessary to confirm their efficacy as therapeutic options against Marburg virus infection (Alsaady et al., 2023). Galidesivir and Favipiravir are antiviral drugs demonstrating potential in treating Marburg virus infection through distinct mechanisms. Galidesivir binds to the viral RNA polymerase, crucial for RNA replication, disrupting its activity and halting virus replication. Favipiravir, or T-705, acts as a nucleoside analog, selectively inhibiting viral RNA-dependent RNA polymerase or inducing fatal mutagenesis upon incorporation into viral RNA. This inhibits viral replication and has shown efficacy in oral administration in a mouse model (Albakri et al., 2023). Combination therapies, which include remdesivir and monoclonal antibodies, hold promise in treating advanced Marburg virus disease. Remdesivir inhibits viral replication by interfering with viral RNA synthesis through the inhibition of viral RNA-dependent RNA polymerase, causing delayed chain termination and template-mediated inhibition mechanisms (Cross et al., 2021). Meanwhile, monoclonal antibodies bind to the virus, preventing further infection and neutralizing it. When used together, these therapies synergize, enhancing overall antiviral activity. This combination treatment has been demonstrated to extend the therapeutic window and provide significant protection in a non-human primate model of Marburg virus disease, particularly when initiated at a critical point in disease progression (UTMB Health, 2021). However, these treatments are still under research and not established for Marburg virus. Direct-acting antivirals (DAAs) target specific steps in the viral life cycle by directly inhibiting essential viral enzymes or proteins. For instance, they can disrupt viral replication by targeting non-structural proteins. While predominantly discussed in the context of hepatitis C virus (HCV), DAAs for Marburg virus are likely to function similarly, aiming to inhibit viral enzymes crucial for genetic material replication. This may involve blocking the viral RNA-dependent RNA polymerase or other key enzymes. However, their application to Marburg virus treatment is still under investigation, necessitating further research to establish efficacy and safety (Teoh et al., 2020; Cross et al., 2021). Tilorone and Quinacrine demonstrate potential in treating Marburg virus through their antiviral properties. Tilorone is believed to induce interferon production, a crucial component of the body’s immune response against viral infections. Its ability to penetrate the blood–brain barrier could be advantageous for treating central nervous system-involved viruses. Quinacrine’s antiviral activity likely stems from its lysosomotropic properties, altering cellular pH and disrupting organelle function crucial for viral replication. Additionally, its binding to the Ebola virus glycoprotein suggests a potential mechanism of action against Marburg virus. While these mechanisms suggest promise, further investigation is needed to determine their efficacy and safety for treating Marburg virus (Puhl et al., 2021). However, licensed medical countermeasures are currently unavailable, and the development of effective treatments is ongoing (Kortepeter et al., 2020). Small molecule inhibitors targeting the Marburg virus function through diverse mechanisms aimed at hindering the virus’s infection and replication process. These mechanisms include direct binding to specific regions of the Marburg virus glycoprotein (GP), such as the internal fusion loop or the HR2 domain, thereby impeding the virus’s fusion with host cells. Additionally, some inhibitors are capable of being trapped in the lysosome, intensifying their exposure within this cellular organelle and augmenting viral inhibition, owing to the lysosome’s acidic environment. Furthermore, certain inhibitors prevent virus-host interactions by obstructing the proteins in host cells that viruses exploit during the late stages of infection. These findings underscore the potential of small molecule inhibitors as viable therapeutic options against the Marburg virus (Edwards and Basler, 2019; Schafer et al., 2021). These inhibitors demonstrate good potency and low cytotoxicity, providing insights for potential antiviral therapeutics (Edwards and Basler, 2019; Schafer et al., 2021). Screening studies have identified various drugs with MARV entry-specific inhibition and synergistic effects on inhibiting viral entry (Cheng et al., 2015; Zhang et al., 2020; Schafer et al., 2021). Additionally, compounds with potent inhibitory activity against both Ebola and Marburg viruses have been identified (Wang et al., 2021) (Table 1).

**Table 1:** Promising therapeutic strategies for Marburg virus infection.

S. No.	Compound	Mechanism of action	Experimental validation	References
1	Estradiol benzoate	Inhibition of VP35 protein, disrupting viral replication and immune evasion	Necessary for confirming therapeutic efficacy	Health NTDI (2023)
2	INVEGA (Paliperidone)	Inhibition of VP35 protein, disrupting viral replication and immune evasion	Necessary for confirming therapeutic efficacy	Health NTDI (2023)
3	Galidesivir	Binds to viral RNA polymerase, halting RNA replication	Efficacy demonstrated in mouse model	Alsaady et al. (2023)
4	Favipiravir (T-705)	Acts as a nucleoside analog, inhibiting viral RNA-dependent RNA polymerase	Efficacy demonstrated in mouse model	Alsaady et al. (2023)
5	Remdesivir	Interferes with viral RNA synthesis by inhibiting RNA-dependent RNA polymerase	Demonstrated efficacy in non-human primate model	Albakri et al. (2023) and Cross et al. (2021)
6	Monoclonal Antibodies	Bind to virus, preventing infection and neutralizing it	Demonstrated efficacy in non-human primate model	Cross et al. (2021)
7	Tilorone	Believed to induce interferon production, enhancing immune response against viral infections	Further investigation needed for efficacy and safety	Teoh et al. (2020)
8	Quinacrine	Lysosomotropic properties alter cellular pH, potentially disrupting viral replication; binds to GP protein	Further investigation needed for efficacy and safety	Teoh et al. (2020)
9	Small Molecule Inhibitors	Direct binding to Marburg virus glycoprotein, lysosome trapping, prevention of virus-host interactions	Further investigation needed for efficacy and safety	Kortepeter et al. (2020) and Schafer et al. (2021)

## 3 Exploring molecular targets and therapeutic approaches

Research on Marburg virus assembly and budding emphasizes the pivotal role of viral proteins, particularly VP40, which drives the process (Martin et al., 2018; Gordon et al., 2019). This involves hijacking the host cytoskeleton and utilizing ubiquitin ligases, ESCRT proteins, and calcium-dependent molecules. Although numerous studies aim to inhibit this process, no approved medications exist yet (Hartlieb and Weissenhorn, 2006; Welsch et al., 2010; Kajihara et al., 2012). Nucleic acid antisense therapeutics, like antisense oligonucleotides (ASOs), have shown potential in inhibiting Marburg virus protein expression and release by targeting viral RNA (Spurgers et al., 2008; Tarn et al., 2021). Despite challenges in stability and delivery, recent advancements enhance their efficacy, making them promising for Marburg virus treatment (Cross et al., 2018; Reza et al., 2021). RNA interference (RNAi) holds promise in Marburg virus treatment, with small interfering RNA (siRNA) demonstrating efficacy in animal models (Ursic-Bedoya et al., 2014). Despite promising results, further research and clinical trials are necessary to evaluate their safety and effectiveness in humans (Ursic-Bedoya et al., 2014; Ye et al., 2023). Host-targeting antivirals, such as T-705 (favipiravir) and remdesivir, show potential against Marburg virus through preclinical investigations (Alsaady et al., 2023; Srivastava et al., 2023). However, no licensed medical countermeasures are available, necessitating further research and clinical trials for effective treatment development (Kortepeter et al., 2020; van Eijk et al., 2023). Interferons and interferon stimulators are under investigation for Marburg virus treatment, considering the virus’s evasion of interferon responses. Further research is required to ascertain their potential effectiveness (Valmas et al., 2010; Valmas and Basler, 2011). Cytokine and chemokine modulators’ potential for Marburg virus treatment warrants further investigation despite limited current information (Bixler and Goff, 2015; Zhu et al., 2018). Elevated expression of IL-6 in MARV-infected primates suggests a role for these molecules, necessitating additional research (Guito et al., 2021; Lu et al., 2022). Broad-spectrum antivirals, such as remdesivir and favipiravir, demonstrate therapeutic efficacy against Marburg virus, although specific treatments are lacking (Cross et al., 2021; Hickman et al., 2022). Combination therapy, including monoclonal antibodies and small-molecule antivirals, shows promise in managing the disease (Zhu et al., 2018; Albakri et al., 2023). Antibody-based therapies, including monoclonal and polyclonal antibodies, such as REGN-EB3 and mAb114, reduce mortality in Marburg virus disease patients. Convalescent plasma containing polyclonal antibodies also holds potential for treatment (Cross et al., 2018; Hargreaves et al., 2021).

## 4 Progressive strategies for combatting Marburg virus: from bench to bedside

Marburg virus disease (MVD) encompasses both preclinical and clinical evaluation of potential therapeutics and vaccines, with promising approaches including immunotherapeutic, small molecule antivirals, and monoclonal antibodies (Kortepeter et al., 2020; Cross et al., 2022). Various vaccine platforms are also under study, with some, like cAd3-Marburg, showing efficacy in Phase I clinical trials. Additionally, T-705 (favipiravir), a broad-spectrum antiviral, has demonstrated effectiveness against Marburg virus in preclinical studies, highlighting its potential as a treatment option. These developments are crucial given the lack of approved vaccines or therapeutics for MVD (Zhu et al., 2018; NIH, 2023; Srivastava et al., 2023). Evaluation of antiviral treatments for Marburg virus involves both *in vitro* and *in vivo* models. For instance, studies have shown T-705’s effectiveness in reducing viral replication and infectious viral loads in mice infected with MARV (Zhu et al., 2018). Furthermore, the establishment of a bioluminescent imaging mouse model for Marburg virus allows real-time analysis of infection processes without sacrificing hosts, facilitating the evaluation of various treatments. Animal models including mice, guinea pigs, and nonhuman primates have been instrumental in understanding disease pathogenesis and evaluating potential treatments and vaccines (Bente et al., 2009; Lei et al., 2020; Srivastava et al., 2023). Phase I and II clinical trials for Marburg virus drugs have demonstrated safety, dose-finding, and proof-of-concept. Several vaccine candidates have shown safety and immunogenicity in healthy adult participants. Phase III trials are in development to assess vaccine efficacy (Cross et al., 2022; Srivastava et al., 2024). Ongoing research efforts in both preclinical and clinical settings aim to develop effective preventive vaccines and treatments for Marburg virus disease, addressing the urgent need for medical countermeasures against this highly infectious and severe illness.

## 5 Challenges and future directions

While current treatments for Marburg virus disease leave much to be desired, a wave of promising antiviral strategies is surging through the development pipeline. This mini review dives into these novel approaches, dissecting small molecule inhibitors, nucleic acid therapies, and immune modulators vying to tackle the virus at its core. We then navigate the treacherous waters of clinical trials, highlighting the hurdles of safety, efficacy, and resistance. Recognizing the crucial role of rapid diagnostics and public health preparedness, we chart a course toward equitable access and outbreak readiness. Looking beyond antivirals, we propose venturing into immunomodulatory therapies and unravelling the mysteries of viral reservoirs. By embracing these future directions, we can rewrite the narrative of Marburg, transforming it from a terrifying threat to a story of human ingenuity and triumph.

## 6 Conclusion

The battle against Marburg virus disease is far from over, but the tides are turning. With a burgeoning arsenal of novel antiviral strategies in the pipeline, we are no longer at the mercy of this deadly pathogen. Small molecule inhibitors, nucleic acid therapies, and immune modulators are wielding their weapons against the virus, disrupting its replication, and bolstering our defences. Yet, the path to victory is fraught with challenges. Clinical trials, with their stringent safety and efficacy demands, stand as gatekeepers, ensuring only the most potent weapons pass through. The Specter of drug resistance looms large, urging us to develop combination therapies and remain vigilant. To truly conquer Marburg, we must equip ourselves with rapid diagnostic tools, ensuring swift intervention. Public health preparedness, built on a foundation of education, infrastructure, and global collaboration, will be our shield against future outbreaks. But our ambitions must not be confined to mere defence. We must venture beyond antivirals, exploring the frontiers of immunomodulatory therapies that empower our own immune system to combat the invader. Unravelling the secrets of viral reservoirs, the hidden sanctuaries where Marburg lurks, could be the key to severing its transmission chain. This is not just a medical pursuit; it is a testament to the human spirit, a refusal to succumb to fear and despair. The future is uncertain, but with unwavering resolve and a united front, we can emerge victorious from this invisible war.

## Author contributions

ShS: Conceptualization, Data curation, Investigation, Methodology, Supervision, Validation, Writing – original draft, Writing – review & editing. SK: Writing – original draft, Writing – review & editing. SA: Writing – original draft, Writing – review & editing. SaS: Funding acquisition, Resources, Writing – original draft, Writing – review & editing. JS: Writing – original draft, Writing – review & editing. ST: Writing – original draft, Writing – review & editing.
